# The quality of maternal nutrition and infant feeding counselling during antenatal care in South Asia

**DOI:** 10.1111/mcn.13153

**Published:** 2021-02-07

**Authors:** Harriet Torlesse, Rukundo K. Benedict, Hope C. Craig, Rebecca J. Stoltzfus

**Affiliations:** ^1^ Nutrition Section UNICEF Regional Office for South Asia Kathmandu Nepal; ^2^ Division of Nutritional Sciences Cornell University Ithaca New York USA; ^3^ The DHS Program ICF International Rockville Maryland USA

**Keywords:** antenatal care, breastfeeding, counselling, maternal nutrition, South Asia

## Abstract

Antenatal care (ANC) provides a platform to counsel pregnant women on maternal nutrition and to prepare the mother to breastfeed. Recent reviews suggest that gaps in the coverage and quality of counselling during pregnancy may partly explain why services do not consistently translate to improved behavioural outcomes in South Asia. This scoping literature review collates evidence on the coverage and quality of counselling on maternal nutrition and infant feeding during ANC in five South Asian countries and the effectiveness of approaches to improve the quality of counselling. Coverage data were extracted from the most recent national surveys, and a scoping review of peer‐reviewed and grey literature (1990–2019) was conducted. Only Afghanistan and Pakistan have survey data on the coverage of counselling on both maternal nutrition and breastfeeding, nine studies described the quality of counselling and three studies assessed the effectiveness of interventions to improve the quality of services. This limited body of evidence suggests that inequalities in access to services, gaps in capacity building opportunities for frontline workers and the short duration and frequency of counselling contracts constrain quality, while the format, duration, frequency and content of health worker training, together with supportive supervision, are probable approaches to improve quality. Greater attention is needed to integrate indicators into monitoring and supervision mechanisms, periodic surveys and programme evaluations to assess the status of and track progress in improving quality and to build accountability for quality counselling, while research is needed to understand how best to assess and strengthen quality in specific settings.

**Key messages**

- Despite significant investments in developing the capacity of frontline workers to provide counselling on maternal nutrition and infant feeding during antenatal care (ANC), there are evidence gaps in the quality of counselling and effectiveness of approaches to improve quality in South Asia.
- Inequalities in access to services, gaps in capacity building opportunities for frontline workers and the short duration and frequency of counselling contacts constrain the quality of counselling.
- The format, duration, frequency and content of health worker training and supportive supervision are probable approaches to improve quality.
- Greater attention is needed to integrate indicators of quality counselling into monitoring and supervision mechanisms, periodic surveys and programme evaluations, while research is needed to understand how best to assess and strengthen quality in specific settings.

## 1 INTRODUCTION

Antenatal care (ANC) enables health care professionals to prevent, identify and treat conditions that may threaten the health of a pregnant woman and her fetus or newborn (Carroli, Rooney, & Villar, 2001). Maternal underweight, obesity and micronutrient deficiencies can endanger the survival, health and well‐being of pregnant women, as well as the growth, development and survival of their infants (Black et al., 2013). For this reason, the World Health Organization's 2016 ANC guidelines include recommendations to counsel pregnant women on healthy eating and keeping physically active, educate pregnant women in undernourished populations on increasing energy and protein intake and encourage women at risk of micronutrient deficiencies to take micronutrient supplements (WHO, 2016). In addition, the recommendations highlight the importance of the quality of care for a positive pregnancy experience. ANC also provides an opportunity to prepare the mother to initiate breastfeeding immediately after delivery and exclusively breastfeed for 6 months (WHO, 2018).

There has been a rapid expansion in the number of countries that offer nutrition counselling to pregnant women as a standard component of ANC (UNICEF, 2019). These services are valued in South Asia where maternal undernutrition is one of the main reasons underlying the region's high prevalence of child wasting (15%) and stunting (34%) (Torlesse & Aguayo, 2018; UNICEF, WHO, and World Bank Group, 2019). An estimated one in two women are anaemic, one in 10 South Asia women have a low stature (<145 cm), one in five have a low body mass index (<18.5 kg m^2^) and one in four infants are born with a low birthweight (<2.5 kg) (Goudet, Murira, Torlesse, Hatchard, & Busch‐Hallen, 2018; Stevens et al., 2013; UNICEF & WHO, 2019). South Asia is performing relatively better than other regions on breastfeeding, but even so, 58% of newborns are not breastfeed immediately after birth and 41% of infants aged less than 6 months are not exclusively breastfed (UNICEF, 2016).

Recent reviews have suggested that gaps in the coverage and quality of nutrition counselling during pregnancy may partly explain why counselling services do not consistently translate to improved dietary intake, consumption of micronutrient supplements and infant feeding practices in South Asian countries (Benedict, Craig, Torlesse, & Stoltzfus, 2018; Goudet et al., 2018). To understand the magnitude of these gaps and how to address them, this study reviews data and literature from South Asia on the coverage and quality of counselling services on maternal nutrition and infant feeding during ANC and on the effectiveness of programmes, interventions and approaches to improve the quality of counselling.

## 2 METHODS

This study examines evidence from the five largest South Asian countries (Afghanistan, Bangladesh, India, Nepal and Pakistan) on the (i) coverage of counselling on maternal nutrition and infant feeding practices given to pregnant women during ANC; (ii) quality of counselling on maternal nutrition and infant feeding practices given to pregnant women during ANC; and (iii) effectiveness of programmes, interventions and approaches to improve counselling on maternal nutrition and infant feeding during ANC. The maternal nutrition practices include meal frequency, quantity of food intake, consumption of nutritious/diverse foods, adherence to intake of iron, folic acid and multiple micronutrient supplements during pregnancy. The infant feeding practices include the early initiation of breastfeeding (EIBF) immediately after delivery, avoidance of prelacteal feeds and exclusive breastfeeding (EBF) for 6 months.

### 2.1 Coverage of counselling

Data on the coverage of maternal nutrition and infant feeding counselling during ANC was compiled from the most recent nationally representative surveys including five Demographic and Health Surveys (Afghanistan 2013, Bangladesh 2014, India 2015–2016, Nepal 2016 and Pakistan 2017–2018) and two national nutrition surveys (Afghanistan National Nutrition Survey 2015 and Bangladesh Child and Mother Survey 2012).

### 2.2 Quality of counselling

#### 2.2.1 Conceptual framework

We developed a conceptual framework that describes factors associated with the quality of counselling services at the levels of the health facility, community, household and individual (Figure S1). Based on existing literature that describes the features of ANC that influence quality of counselling (Bitton et al., 2017; Donabedian, 1980; Hulton, Matthews, & Stones, 2000), we identified five key factors: accessibility to services, resource availability, environmental readiness, service provider readiness and interactions between service provider and client. A set of indicators, derived from service provider guidelines, training manuals and job aides on counselling (Alive & Thrive, 2013; Gavin et al., 2014; IYCN, 2012; UNICEF, 2011; WHO, 2012, 2015), were defined for each of the five factors (Table 1).

**TABLE 1:** Indicators of the quality of counselling on maternal nutrition and infant feeding during antenatal care

Factor	Indicators
(A) Accessibility Geographic, financial, sociocultural and temporal access to counselling services and timeliness, frequency and duration of counselling	A1. Timeliness of visit, for example, gestational age of mother matches information/counselling received A2. Frequency and duration of counselling A3. Cost of accessing counselling service, for example, transportation, user fees and opportunity costs A4. Distance to counselling services A5. Sociocultural barriers or enablers to women and family members accessing counselling services
(B) Resource availability Sociocultural appropriateness of counselling messages and availability of hardware and skilled health workers	B1. Socioculturally adapted maternal nutrition and infant feeding messages B2. Socioculturally appropriate job aids available B3. Knowledgeable and skilled health workers available to counsel clients
(C) Environmental readiness Physical, sociocultural and operational preparedness to deliver counselling	C1. Pleasant, accessible physical environment, for example, privacy, toilets and clean water C2. Welcoming atmosphere, for example, non‐discriminatory, safe and client feels comfortable talking with health worker C3. Health workers present at ANC location, for example, adequate number of health workers that provide ANC services at facility regularly C4. Minimized waiting times, for example, client satisfaction with waiting time C5. Posters and other materials visible and available for clients
(D) Provider readiness Appropriately skilled, motivated and supervised health workers	D1. Adequate and appropriate health worker training D2. Frequency of refresher training for health worker D3. Health worker motivation D4. Health worker supervision frequency and quality
(E) Service provider and client interactions Quality of interactions between client and health worker in the delivery of counselling services	E1: Heath worker establishes and maintains a trusting environment and builds rapport with client (e.g., greets client, uses verbal and non‐verbal responses that show interest, speaks in a respectful/polite manner and takes time) E2: Health worker uses listening and learning skills to assess the client's needs and personalizes the discussions accordingly E3: Health worker provides information/advice on optimal practices and explains why they are important in a way that is easily understood and retained by the client E4: Health worker works interactively with the client to address concerns and questions, give practice support, build confidence and establish a plan to change behaviours E5: Health worker confirms the client's understanding E6: Health worker involves other family members (if present) E7: Client satisfaction with counselling services

Abbreviation: ANC, antenatal care.

#### 2.2.2 Study framework

A scoping methodology framework proposed by Arksey and O'Malley (2005) was used. It involved a five‐stage review process: (i) identification of the research objectives, (ii) identification of relevant studies, (iii) section of studies, (iv) mapping of the data and (v) organization, summary and reporting of the results.

#### 2.2.3 Literature search

A PICOT search strategy, including the elements of population, intervention, comparison, outcomes and time, was used. PubMed was searched to identify research articles published between January 1990 and May 2019. Search terms were applied with various Boolean operators for country location, target group, ANC and counselling, maternal nutrition and infant feeding and date of publication (Figure S2). In addition, grey literature published between January 1990 and August 2019 on programme evaluations was sourced from experts and organizational websites, including the WHO Library Database, Human Resources for Health Global Resource Center and the International Initiative for Impact Evaluation.

#### 2.2.4 Study selection

Eligibility criteria included the study location (Afghanistan, Bangladesh, India, Nepal and Pakistan), year published (1990–2019), at least one measured indicator of the quality of counselling, study design, English language and full‐text availability. Duplicate citations were removed, and titles and abstracts were screened to identify relevant studies. Two researchers independently screened the full texts of all potentially relevant articles to assess eligibility. Any discrepancies between reviewers were resolved through discussion and consultation with a third researcher.

#### 2.2.5 Synthesis of data

For studies that examine the quality of counselling on maternal nutrition and infant feeding during ANC, data were synthesized by source and country; study setting, design and sample; maternal nutrition and infant feeding practices examined; and reported results for each indicator of the counselling quality. For studies that examine the effectiveness of programmes, interventions and approaches to improve to improve counselling on maternal nutrition and infant feeding during ANC, data were synthesized by source and country; study design, subjects, intervention group and comparison group; maternal nutrition and infant feeding practices examined; and reported results for each indicator of counselling quality. Effectiveness to improve counselling was defined as measured improvements in any of the indicators of the quality of counselling in the intervention group compared with the comparison group.

### 2.3 Ethical considerations

Ethical approval was not required for this study as it used data from existing reports and articles.

## 3 RESULTS

After excluding duplicates, 924 unique articles were screened, and 12 studies were selected (Figure S3), including 10 peer‐reviewed articles and two grey literature reports. Findings are reported by evidence on the quality of counselling on maternal nutrition and infant feeding (nine studies) and effectiveness to improve the quality of counselling (three studies).

### 3.1 Coverage of counselling services during ANC

Data extracted from national survey reports on the coverage of maternal nutrition and infant feeding counselling during ANC are provided in Table 2 together with data on the coverage of four or more ANC visits during pregnancy. All five countries have survey data on ANC coverage; however, only two countries have data on the coverage of counselling on maternal dietary intake (Afghanistan and Pakistan), and three countries have data on the coverage of breastfeeding counselling (Afghanistan, India and Pakistan).

**TABLE 2:** Proportion of women aged 15–49 years who received counselling on dietary intake and breastfeeding during antenatal care during their most recent pregnancy

		Four or more antenatal care visits	Dietary intake^a^	Breastfeeding
Afghanistan	National Nutrition Survey, 2013	17.8	47.7%	15.8% (exclusive)
	Demographic and Health Survey, 2015	‐	‐	‐
Bangladesh	Demographic and Health Survey, 2014	31.2	‐	‐
India	National Family Health Survey‐4, 2015–2016	51.2	‐	80.4% (breastfeeding)
Nepal	Demographic and Health Survey, 2016	69.4	‐	‐
Pakistan	Demographic and Health Survey, 2017–2018	51.4	69.6%	52.2% (early initiation) 54.3% (exclusive)

^a^ Includes counselling of pregnant women during antenatal care (ANC) on one or more of the following: meal frequency, quantity of food intake and consumption of balanced diet or nutritious/diverse foods during pregnancy.

Country comparisons of counselling coverage are only possible for Afghanistan and Pakistan, which have data on the same indicators. The proportion of women who received counselling on dietary intake was almost 50% higher in Pakistan than Afghanistan (70% vs. 48%) whereas counselling on EBF was more than three times higher in Pakistan than Afghanistan (54% vs. 16%). A possible explanation for these differences is that Pakistan has a stronger network of health workers to provide counselling to women during pregnancy, including its network of community‐based lady health workers (LHWs). In both Afghanistan and Pakistan, the coverage of counselling on EBF is lower than maternal dietary intake (16% vs. 48% in Afghanistan and 54% vs. 70% in Pakistan). This indicates that the primary health care services in both countries are giving relatively greater attention to promoting a healthy diet during pregnancy than to preparing pregnant women to exclusively breastfeed.

The coverage of four or more ANC visits during pregnancy was similar to the coverage of counselling on EBF in Afghanistan (18% vs. 16%) and Pakistan (51% vs. 52%). However, there were large differences in the coverage of four or more ANC visits and counselling on dietary intake in Afghanistan (18% vs. 48%) and Pakistan (51% vs. 70%) and in the coverage of four or more ANC visits and breastfeeding counselling in India (51% vs. 80%). This suggests that these countries are utilizing other service delivery platforms in addition to ANC services to provide counselling on dietary intake or breastfeeding to women during pregnancy, such as community‐based workers.

### 3.2 Quality of counselling on maternal nutrition and infant feeding

#### 3.2.1 Characteristics of included studies

The nine studies included four that were conducted in India (Avula et al., 2015; Dhandapany, Bethou, Arunagirinathan, & Ananthakrishnan, 2008; Pricilla et al., 2017; Singh, Pallikadavath, Ram, & Ogollah, 2012), three in Pakistan (Dykes, Lhussier, Bangash, Zaman, & Lowe, 2012; Mahar et al., 2012; Majrooh Majrooh, Hasnain, Akram, Siddiqui, & Memon, 2014), one in Bangladesh (Huda et al., 2018) and Nepal (McPherson et al., 2010), and no eligible studies were conducted in Afghanistan. All studies examined both maternal nutrition and infant feeding practices except Dhandapany et al. (2008), which reported on infant feeding only. The study designs included four cross‐sectional studies (Avula et al., 2015; Dhandapany et al., 2008; Mahar et al., 2012; Singh et al., 2012), one prospective cohort study (Pricilla et al., 2017), two qualitative studies (Dykes et al., 2012; McPherson et al., 2010) and two mixed methods studies (Huda et al., 2018; Majrooh et al., 2014). The studies collectively described four out of five factors of quality and 13 out of 24 indicators; no studies examined indicators of environmental readiness. A summary of the included studies is provided in Table 3, and Table S1 lists the indicators on quality of counselling for each of the included studies.

**TABLE 3:** Summary of studies examining the quality of counselling on maternal nutrition and infant feeding during antenatal care

Source and country	Study setting, design and sample	Maternal nutrition and infant feeding practices examined	Results
Avula et al. (2015) India	Setting: Essential nutrition interventions are provided to women and children during monthly Village Health Nutrition Days at Anganwadi centres (AWCs) or through home visits. These services are delivered through the Integrated Child Development Services (ICDS) and National Rural Health Mission (NRHM) by frontline workers (FLWs) of these two programmes: Anganwadi workers (AWWs) from ICDS and accredited social health activists (ASHAs) and auxiliary nurse midwives (ANMs) from NRHM Design: Cross‐sectional study Sample: 1136 mothers of children aged 0–23 months and 717 FLWs	Maternal nutrition: Maternal dietary intake (diversity and additional food intake); iron and folic acid (IFA) supplementation Infant feeding: Early initiation of breastfeeding (EIBF)	Availability of knowledgeable health workers: • 56% of AWW, 56% of ASHA and 66% of ANMs had knowledge on importance of diverse diet during pregnancy (B3). • 47% of AWW, 55% of ASHA and 53% of ANMs had knowledge on need for additional food intake during pregnancy (B3). • 69% of AWW, 54% of ASHA and 56% of ANMs had knowledge on need of IFA supplements during pregnancy (B3). • 99% of AWW, 99% of ASHA and 100% of ANMs had knowledge on EIBF (B3). Provision/receipt of advice and counselling: • >80% women received nutrition counselling during ANC (E3). • >75% women received advice about IFA supplements during pregnancy (E3).
Dhandapany et al. (2008) India	Setting: Rural pregnant women attending a tertiary hospital for delivery. Most rural women in the locality of the hospital received ANC care from an ANM Design: Cross‐sectional study Sample: 108 primigravida women who had received three or more ANC visits	Infant feeding: EIBF, exclusive breastfeeding (EBF) and avoidance of prelacteal feeding (APF)	Provision/receipt of advice and counselling: • 21% women received counselling on breastfeeding during ANC (E3).
Dykes et al. (2012) Pakistan	Setting: Nutrition Support Program provided by lady health worker (LHW), including education in the form of cookery demonstrations offered weekly, food assistance (milk powder, rice and pulses) provided to women and nutritional supplements provided to women identified as malnourished. Rural, facility‐based (Emergency Satellite Hospital) Design: Formative qualitative design (focus group discussions) Sample: 16 LHWs	Maternal nutrition: Maternal dietary intake (diversity and additional intake) Infant feeding: Breastfeeding information	Socioculturally adapted job aids available: • Cookery demonstrations offered low‐tech, low‐cost intervention using local foods that engaged local women who were pleased to participate (B2). Include other family members into ANC counselling: • LHWs suggest that men should be involved in health education initiatives because of their role in decision making (E6). Frequency of refresher training for health worker: • LHWs reported that further training of LHWs is needed (D2).
Huda et al. (2018) Bangladesh	Setting: Selected villages with low socio‐economic conditions in two unions in Kendua subdistricts of Netrokona district Design: Mixed methods study involving cross‐sectional survey, focus group discussions and in‐depth interviews Sample: 275 women who were pregnant or had a birth in the last 6 months	Maternal nutrition: Amount and frequency of food intake during pregnancy Infant feeding: EBF	Acceptability and perceived appropriateness of mobile phone counselling: • 95% women were satisfied with the direct counselling through mobile phone and answers provided to their queries (B2 and E7). • 22% women reported that the frequency of the biweekly calls was not sufficient (A2). • Most women understood the content of the counselling and felt the information provided was very important and beneficial for both mother and child (E7). • Two thirds missed at least one counselling call because of household responsibilities or difficulties in charging the mobile phones (A5).
Mahar et al. (2012) Pakistan	Setting: Urban private and public sector hospitals providing antenatal care services Design: Cross‐sectional study Sample: 216 pregnant women	Maternal nutrition: Dietary intake Infant feeding: Breastfeeding	Frequency and duration of counselling: • Average duration of communication between pregnant woman and health care provider on all topics was 3 min in public hospital and 8 min in private hospital (A2). Provision/receipt of advice and counselling: • Proportion of women receiving advice on diet and nutrition was 86% in private hospital and 53% in public settings (A5 and E3). • No antenatal clients in private or public hospitals received any advice or counselling on breastfeeding (E3).
Majrooh et al. (2014) Pakistan	Setting: ANC services delivered by LHWs in rural health centres (RHCs) and basic health units (BHU) Design: Cross‐sectional study and qualitative research methods Sample: 17 BHU and two RHCs selected from each of nine districts. Focus group discussions and in‐depth interview conducted with clients, providers and health managers	Maternal nutrition: Dietary intake Infant feeding: Breastfeeding	Provision/receipt of advice and counselling: • 63% clients reported counselling on advice on dietary intake (E3). • 6% of clients reported counselling on breastfeeding (E3).
McPherson et al. (2010) Nepal	Setting: Community‐based maternal and neonatal services in rural Nepal Design: Qualitative process evaluation of interpersonal communication delivered by Female Community Health Volunteers (FCHVs) to pregnant women using a flipchart and pictorial booklet that was distributed to clients Sample: Semistructured interviews with four central‐level NFHP staff, nine district‐level and NFHP health officers, eight community health centre staff members, 29 FCHVs and 23 women who had delivered within 3 months of interview, together with family members	Maternal nutrition: Maternal diet Infant feeding: EIBF	Socioculturally adapted maternal nutrition and infant feeding messages and job aids: • Information, content and length were found to be appropriate by clients (B1). • Messages on maternal nutrition were understood by women, but maternal nutrition was not rated as important as messages on danger signs and newborn care (B1). • All family members liked the booklet and found it a helpful resource (B2). Include other family members into ANC counselling: • FCHV involved any senior household members (husbands and mothers‐in‐law) who were present at the FHCV's or client's home during the ANC visit (E6). Health worker motivation and supervision: • FCHVs found it difficult to find time for new tasks, but supervision by facility staff was motivating for some (D3 and D4).
Pricilla et al. (2017) India	Setting: Urban health centre of tertiary teaching medical college and hospital in South India Design: Prospective cohort study Sample: 200 low‐risk pregnant women receiving ANC from a trained nurse midwife, including education on dietary intake and breastfeeding	Maternal nutrition: Dietary advice on iron‐rich foods Infant feeding: Breastfeeding (breast/nipple care advice)	Health worker establishes and maintains a trusting environment and builds rapport with client: • 100% women reported the nurse midwife greeted them (E1). • 100% women reported that the nurse midwife was polite (E1). Health worker provides information/advice on optimal practices: • 98% of women reported receiving diet advice on iron‐rich foods (E3). • 75% of women reported receiving breast/nipple care advice (E3). Health worker works interactively with the client to address concerns and questions: • 99% women reported that the nurse wife addressed her concerns and 95.5% reported that the nurse midwife answered their questions (E4).
Singh et al. (2012) India	Setting: Rural India, with comparisons in the provision of antenatal care drawn between lower level facilities (health subcentres and primary health centres) and higher level facilities (community health centres, rural hospitals, first referral units and hospitals) Design: Cross‐sectional study Sample: District‐level household survey data conducted in 601 districts from 34 states, including 643,944 ever married and 166,260 unmarried women aged 15–49 years	Maternal nutrition: Nutrition during pregnancy Infant feeding: Breastfeeding	Provision/receipt of advice and counselling: • Proportion of women receiving advice on maternal nutrition and breastfeeding during ANC was higher in higher level facilities than lower level facilities (maternal nutrition 68% vs. 66%; breastfeeding 75% vs. 70%) (A5 and E3). • Odds on receipt of advice on maternal nutrition and breastfeeding during ANC were significantly higher among wealthier households than poor households in both lower level and higher level facilities (A5).

Abbreviation: ANC, antenatal care.

#### 3.2.2 Accessibility

Accessibility is defined as geographic, financial, sociocultural and temporal access to counselling services and the timeliness, frequency and duration of counselling. Three studies examined one or more indicators of accessibility (Huda et al., 2018; Mahar et al., 2012; Singh et al., 2012). A study in India found that pregnant women were more likely to receive advice on maternal nutrition and breastfeeding if they belonged to wealthier households and utilized higher level facilities (Singh et al., 2012), whereas in Pakistan, coverage of counselling services was higher among clients who accessed health care from private facilities than public facilities (Mahar et al., 2012), indicating socio‐economic barriers among poorer households. Mahar et al. (2012) reported that ANC counselling interactions in public and private hospitals in urban Pakistan lasted only 3 and 5 min, respectively. A pilot study in Bangladesh assessed the feasibility, acceptability and perceived appropriateness of mobile phone counselling of pregnant women and mothers on maternal nutrition and infant feeding (Huda et al., 2018). It found that 22% of women were not satisfied with the frequency of biweekly counselling and that two thirds of women missed at least one counselling call because of household responsibilities or difficulties in charging their mobile phones.

#### 3.2.3 Resource availability

Resource availability is defined as the sociocultural appropriateness of counselling messages and availability of hardware and skilled health workers. Four studies examined resource availability, including client satisfaction with the types of messages, and job aids and approaches used by health workers and reported positive feedback (Avula et al., 2015; Dykes et al., 2012; Huda et al., 2018; McPherson et al., 2010). In Nepal, pregnant women and other family members appreciated a pictorial booklet that was used by health workers to counsel women and found that the information, contents and length were appropriate and the messages understood (McPherson et al., 2010). In Pakistan, women were pleased to participate in cookery demonstrations that were organized by LHWs to inform and counsel women on dietary quality (Dykes et al., 2012). In Bangladesh, women reported that they were satisfied with direct counselling through mobile phones (Huda et al., 2018), although this was their subjective perception and does not mean that the messages or approaches used to convey the messages were appropriate. A study in India examined the knowledge of different cadres of frontline workers, Anganwadi workers (AWW), accredited social health activists (ASHA) and auxiliary nurse midwives (ANMs) (Avula et al., 2015). It found that the proportion of frontline workers that had accurate knowledge on EBF (99–100%) was greater than on aspects of maternal nutrition, including the importance of a diverse diet (56–66%), additional food intake during pregnancy (47–53%) and iron and folic acid (IFA) supplements during pregnancy (69–56%). There was no consistent relationship between the type of frontline worker and knowledge. However, maternal acquisition of the counselling messages depends both on health workers sufficient knowledge and their ability to effectively transfer this knowledge to mothers (Mbuya, Menon, Habicht, Pelto, & Ruel, 2013).

#### 3.2.4 Provider readiness

Provider readiness is defined as appropriately skilled, motivated and supervised health workers. Only two studies examined indicators on provider readiness (Dykes et al., 2012; McPherson et al., 2010). In Pakistan, LHWs, who provided counselling to pregnant women at community level, reported that they need more frequent refresher training (Dykes et al., 2012), whereas supportive supervision by facility‐based health workers in rural Nepal helped to motivate Family and Child Health Volunteers to ‘work harder’ (McPherson et al., 2010).

#### 3.2.5 Service provider and client interactions

All nine studies examined one or more indicators on the quality of interactions between service provider and client in the delivery of counselling services. Six studies reported on the proportion of clients who received information/advice on optimal practices from health workers; this proportion varied widely according to the subject of the counselling, study setting and the type of health facility. Pregnant women were less likely to receive information or counselling on breastfeeding than on maternal nutrition in studies that made this comparison in Pakistan (Mahar et al., 2012; Majrooh et al., 2014) and urban India (Pricilla et al., 2017) but not in rural India (Singh et al., 2012). Studies in Pakistan found that the counselling coverage was greater in private health facilities than public facilities (Mahar et al., 2012) and higher in greater level facilities than lower level facilities (Pricilla et al., 2017). Other indicators of the interactions between service provider and client were included in only one or two studies, and no studies examined the health workers' listening and learning skills or whether the health worker confirmed the client's understanding. Two studies examined participation by men and other family members in counselling; in Nepal, McPherson et al. (2010) found that Female Community Health Volunteers involve husbands and mothers‐in‐law during ANC visits, whereas Dykes et al. (2012) reported that LHWs suggest husbands should be involved in health education initiatives in Pakistan because of their role in decision making. Huda et al. (2018) explored mobile phone‐based counselling and reported that 95% of women were satisfied with these services.

### 3.3 Effectiveness to improve the quality of counselling

The three included articles (Table 4) relate to studies conducted in India (Baqui et al., 2006) and Bangladesh (Nguyen et al., 2017, 2018); no eligible studies were conducted in Afghanistan, Nepal or Pakistan. The studies examined the impact of programme interventions on indicators of accessibility (Nguyen et al., 2018), resource availability (Baqui et al., 2006; Nguyen et al., 2018), provider readiness (Nguyen et al., 2018) and service provider and client interactions (Baqui et al., 2006; Nguyen et al., 2017, 2018); no studies reported on indicators of environmental readiness. Table S2 lists the indicators on quality of counselling for each of the included studies.

**TABLE 4:** Effectiveness of programmes, interventions and approaches to improve quality of counselling on maternal nutrition and infant feeding during antenatal care

Source and country	Study design, subjects, intervention group (IG) and comparison group (CG)	Maternal nutrition and infant feeding practices examined	Results (IG vs. CG)
Baqui et al. (2006) India	Design: Quasi‐experimental (baseline and endline cross‐sectional surveys) Subjects: 909 frontline workers (FLWs) including auxiliary nurse midwives (ANMs), Anganwadi workers (AWWs) and change agents (CAs) IG: As part of the Integrated Nutrition and Health Project II, ANMs and AWWs were trained to deliver maternal and child health (MCH) services, and a new cadre of volunteers called CAs were recruited and trained. Maternal care intervention included at least three ANC visits with the provision of and information about IFA supplementation and information on maternal nutrition and rest. Newborn care interventions included EIBF and EBF. CG: FLW received routine training and pregnant women received services provided by Integrated Child Development Services (ICDS) and Ministry of Health.	Maternal nutrition: IFA supplementation Infant feeding: EIBF and EBF	Increase in FLW knowledge between baseline and endline (B3) • Greater increase in % ANW with EBF knowledge in IG than CG: +13 pp versus +1 pp • Greater increase in % AWW with EBF knowledge in IG than CG: +14 pp versus +4 pp • Increase in % CA with EIBF knowledge (IG only): +9 pp • Increase in % CA with IFA knowledge (IG only): +15 pp Proportion of pregnant women receiving counselling during ANC home visits by FLW (E3): • Counselling on IFA by ANM: Smaller increase in IG than CG: +11 pp versus 14 pp. • Counselling on IFA by AWW: Larger increase in IG than CG: +34 pp versus 21 pp. • Counselling on IFA by CA (IG only): Increase: +29 pp. • Counselling on EIBF by ANM: Greater increase in IG than CG: +22 pp versus 2 pp. • Counselling on EIBF by AWW: Greater increase in IG than CG: +29 pp versus 2 pp. • Counselling on EIBF by CA (IG only): Increase: +20 pp. • Counselling on EIBF by CA (IG only): Increase: +20 pp. • Counselling on EBF by ANM: Greater increase in IG than CG: +30 pp versus 6 pp. • Counselling on EBF by AWW: Greater increase in IG than CG: +33 pp versus 4 pp. • Counselling on EBF by CA (IG only): Increase: +22 pp.
Nguyen et al. (2017) Bangladesh	Design: Cluster RCT Subjects: Pregnant or recently delivered women at baseline (*n* = 2000) and at endline (*n* = 2000). Data on dietary intake obtained from a subset of 600 women IG: Intensified interpersonal communication on maternal nutrition, promotion of optimal breastfeeding practices, provision of free IFA and calcium supplements, weight gain monitoring, community engagement and family involvement. CG: Nonintensive package consisting of antenatal care with standard maternal nutrition counselling, which had few visits and much less nutrition content or emphasis.	Maternal nutrition: Dietary diversity during pregnancy, IFA supplements and calcium supplements Infant feeding: EIBF, EBF and avoidance of prelacteal foods	Proportion of women receiving information on maternal nutrition and breastfeeding during pregnancy (E1): Increase between baseline and endline significantly greater in IG than CG for receipt of information on eating at least five food groups (DDE 66.5 pp, *P* < 0.001) and avoidance of prelacteal foods (DDE 16.0 pp, *P* < 0.05) but not for eating additional food, taking IFA supplements or taking calcium supplements, EIBF or EBF.
Nguyen et al. (2018) Bangladesh	Design: Cluster RCT Subjects: 437 FLW and 4000 pregnant women IG: Capacity of FLW was developed to deliver intensified interpersonal communication to pregnant women on maternal nutrition, promote breastfeeding, monitor pregnancy weight gain, distribute supplements and engage family and community members. Capacity development initiatives included hands‐on training, monthly meetings, service delivery support through supplies and job aids (e.g., diet chart and nutrition calendar), supportive supervision and feedback, performance‐based cash incentives and continued refresher training. CG: FLW did not receive any additional capacity development initiatives to deliver nutrition services to pregnant women.	Maternal nutrition: Dietary diversity during pregnancy, IFA supplements and calcium supplements Infant feeding	Score on the quality of FLW training (D1): Increase between baseline and endline greater in IG (2.5 to 4.7) than CG (2.6 to 2.4). DDE 2.42 (95% CI: 1.34, 3.50). Months since FLW last received refresher training on maternal nutrition and infant feeding (D2): Increase between baseline and endline smaller in IG (0.47 to 0.6) than CG (0.31 to 1.2). DDE −0.71 (95% CI: −1.28, −0.14). Score of FLW knowledge of maternal nutrition and infant feeding (B3): Increase between baseline and endline greater in IG (6.5 to 7.5) than CG (6.0 to 5.9). DDE 1.21 (95% CI: 0.42, 2.00). Duration (minutes) that FLW spent discussing maternal nutrition and infant feeding during home visit (A2): Decrease between baseline and endline greater in IG (16.4 to 16.2) than CG (14.9 to 16.9). DDE −3.21 (95% CI: −0.641, −0.01). Score of quality of counselling (as defined by the number of messages women received on maternal nutrition and infant feeding) (E3): Increase between baseline and endline greater in IG (3.8 to 5.6) than CG (3.8 to 3.9). DDE 1.6 (95% CI: 0.70, 2.49). Score of FLW perceptions on the quality of supervision (D4): Difference between baseline and endline similar in IG (8.9 to 8.4) and CG (8.4 to 8.4). DDE −0.28 (95% CI: −0.94, 0.38).

Abbreviations: ANC, antenatal care; EBF, exclusive breastfeeding; EIBF, early initiation of breastfeeding; IFA, iron and folic acid.

Baqui et al. (2006) report on the findings of a programme evaluation (quasi‐experimental study) of CARE‐India's newborn health intervention. In the programme areas, ANMs and AWWs were trained to deliver information and counselling on maternal nutrition and infant feeding, and a new cadre of volunteers called change agents were recruited and trained. Between baseline and endline, the increase in knowledge of ANW and AWW on EBF and receipt of counselling on IFA supplementation, EIBF and EBF was greater in the intervention area compared with the comparison area where ANMs and AWWs received routine training and no change agents were recruited.

In Bangladesh, a cluster randomized control trial evaluated the effect of providing nutrition‐focused ANC compared with standard ANC on maternal dietary diversity, micronutrient supplement intake and early breastfeeding practices (Nguyen et al., 2017, 2018). In the intervention areas, the capacity of frontline workers was developed to deliver intensified interpersonal communication to pregnant women on maternal nutrition, monitor pregnancy weight gain, distribute free IFA and calcium supplements, promote breastfeeding and engage family and community members. Capacity development initiatives included hands‐on training, monthly meetings, service delivery support through supplies and job aids (e.g., diet chart and nutrition calendar), supportive supervision and feedback, performance‐based cash incentives and continued refresher training. In comparison areas, the frontline workers did not receive any additional capacity development initiatives, and a nonintensive package consisting of ANC with standard maternal nutrition counselling was delivered, which had fewer visits and less nutrition content. Compared with the comparison areas, frontline workers in the intervention areas received better quality training, received more frequent fresher training and had superior knowledge of maternal nutrition and infant feeding at endline (Nguyen et al., 2018). The increase between baseline and endline in the proportion of women receiving information was significantly greater in the intervention group than the comparison group for the receipt of information on eating at least five food groups and avoidance of prelacteal foods but not for eating additional food, taking IFA supplements or taking calcium supplements, EIBF or EBF (Nguyen et al., 2017). Overall, the increase between baseline and endline in the number of messages on maternal nutrition and breastfeeding received by women was greater in the intervention area than the comparison group (Nguyen et al., 2018). Differences in coverage of service delivery and counselling quality explained a large portion of the variation among villages for consumption of IFA and calcium supplements, whereas differences in the quality of counselling services explained 60% of the programme's impact on women's dietary diversity during pregnancy (Nguyen et al., 2018).

## 4 DISCUSSION

Despite investments across South Asia in building the capacity of health workers to counsel pregnant women on maternal nutrition and infant feeding during ANC (UNICEF, 2019), our review of recent national survey reports and literature published since 1990 has identified that there is little evidence on the coverage or quality of these counselling services or on what works to improve counselling quality in South Asia's five largest countries (Afghanistan, Bangladesh, India, Pakistan and Nepal).

Only Afghanistan and Pakistan have nationally representative survey data on the coverage of counselling on both maternal dietary intake and breastfeeding during ANC. The available data indicates that access to counselling is a problem and varies considerably between countries and that the coverage of counselling on breastfeeding lags behind maternal dietary intake. These coverage indicators were not included in previous rounds of the core DHS questionnaires but were added to most recent Pakistan and India DHS and national nutrition survey in Afghanistan. As global attention expands from measuring ANC coverage to the contents of ANC care (Benova, Tunçalp, Moran, & Campbell, 2018), it is important that counselling on maternal nutrition and breastfeeding be considered with the same value as other interventions when decisions are made on the selection of survey indicators to assess the content of ANC services. The current core DHS questionnaires now include questions on maternal nutrition and breastfeeding counselling (DHS Program, 2019). In addition, there is need to ensure that routine health information systems in all countries include indicators to monitor the coverage of services to counsel pregnant women on dietary intake and breastfeeding.

We developed a conceptual framework and set of 24 indicators to characterize the quality of counselling across five key factors (accessibility to services, resource availability, environmental readiness, service provider readiness and interactions between service provider and client). We used this framework to gather evidence on the quality of counselling on maternal nutrition and breastfeeding and the effect of programmes, interventions and approaches to improve quality in South Asia. The literature searches revealed that most research studies focused on behavioural outcomes (dietary practices, adherence to micronutrient supplements and infant feeding practices) and did not examine the quality of counselling provided to pregnant women and their family members. Only nine studies published since 1990 described at least one indicator of the quality counselling, and only two studies reported on approaches to improve the quality of counselling.

The nine studies on the quality of counselling collectively included evidence on 13/24 indicators; the median number of indicators included each study was only three and no studies examined indicators of environmental readiness. Although this body of evidence provides a limited insight into the quality of counselling in South Asia, several common findings emerged. Inequities in access to counselling services are common in South Asia countries, with poorer households and those utilizing lower level or public health facilities less likely to receive counselling that wealthier households or those utilizing higher level or private facilities (Mahar et al., 2012; Singh et al., 2012), consistent with the findings of global studies on socio‐economic disparities in access to ANC services (Amo‐Adjei, Aduo‐Adjei, Opoku‐Nyamaah, & Izugbara, 2018; Arsenault et al., 2018). The short duration and low frequency of counselling contacts are challenges (Huda et al., 2018; Mahar et al., 2012), whereas client satisfaction with messages, job aids, use of mobile phones and cookery demonstrations tended to be high (Dykes et al., 2012; Huda et al., 2018; McPherson et al., 2010). Information on provider readiness is very limited, with just one study in Pakistan reporting that LHWs seeking greater opportunities for refresher training (Dykes et al., 2012) and a study in rural Nepal reinforcing the value of supportive supervision in motivating frontline workers (McPherson et al., 2010).

We found only three studies that examined approaches to improve the quality of counselling during ANC, and all included a very small number of indicators (range one to five). The most comprehensive study (Nguyen et al., 2018) examined the effect of efforts to build the capacity of frontline workers through hands‐on training, monthly meetings, service delivery support through supplies and job aids, supportive supervision and feedback, performance‐based cash incentives and continued refresher training. It reported significant improvements between baseline and endline in the intervention group relative to comparison group in the quality of training, knowledge of health workers, frequency of refresher training, number of messages given to clients but not the duration of counselling interactions or perceptions of frontline workers on the quality of supervision. With this limited body of evidence, it is not possible to draw firm conclusions on what works to improve the quality of counselling. Nevertheless, the format, duration, frequency and content of health worker training, together with supportive supervision are probable components of interventions that improve the quality of counselling.

These findings show that studies which examine counselling on maternal nutrition and infant feeding during ANC in South Asia rarely focus the quality of counselling and none do so comprehensively. These evidence gaps need greater attention to ensure that poor quality counselling services do not continue unchecked and to guide the design of these services so that they are more impactful on maternal nutrition and infant feeding outcomes. Our framework of indicators on the quality of counselling may help to inform the development of metrics to measure the multidimensional features of the quality of counselling. However, more research is needed to identify indicators that reflect the most impactful aspects of quality in specific settings and to understand the pathways through which the quality of counselling on maternal nutrition and infant feeding can be strengthened. ANC programmes should adapt and integrate quality of counselling indicators into programme monitoring, supportive supervision mechanisms and programme evaluations. A challenge that remains for countries is to find low‐cost, efficient and real‐time methods to assess the quality of counselling. There is also need for advocacy and political will to address disparities in access to quality services to ensure equity in the provision of care.

In line with the scoping review methodology, our review of literature did not grade the quality of evidence. To provide the highest level of evidence on intervention effectiveness, we limited our eligibility criteria to include only randomized controlled trials and quasi‐experimental studies as these designs provide the most precise estimates of the likely effects of an intervention with limited risk of bias. The scarcity of data and information on the coverage, quality and effectiveness of approaches to improve the quality of counselling during ANC is both a limitation and key finding of this study.

## 5 CONCLUSION

ANC provides an important platform to inform and counsel pregnant women and their family members. Our review highlights the shortage of data and evidence on the coverage and quality of counselling on maternal nutrition and infant feeding offered to women during ANC in South Asia and effectiveness of programmes, interventions and approaches to improve quality. Greater attention is needed to integrate appropriate indicators into monitoring and supervision mechanisms, periodic surveys and programme evaluations in order to assess the status and track progress in improving quality, build accountability for quality counselling, and to conduct research to understand what works to improve the quality of counselling.

## CONFLICTS OF INTEREST

The authors declare that they have no conflicts of interest.

## CONTRIBUTIONS

HT, RKB and HCC conceptualized and conducted the analyses. The manuscript was written by HT and edited by RKB, HCC and RJS.

## Supporting information


**Figure S1:** Conceptual framework of factors related to the quality of counselling on maternal nutrition and infant feeding delivered to pregnant women during antenatal care.
Figure S2. Example of search terms applied to the titles and abstracts of articles
Figure S3. Flow chart of the study selection process for eligible studies.
Table S1: Indicators† of counselling quality studied by each of the included studies that examined the quality of counselling on maternal nutrition and infant feeding during antenatal care†
Table S2: Indicators† of counselling quality studied by each of the included studies that examined the effectiveness of programs, interventions, and approaches to improve quality of counselling on maternal nutrition and infant feeding during antenatal care

## Data Availability

The data are presented in the tables and supplementary materials of the article.
