# Supplementary material for: The quality of maternal nutrition and infant feeding counselling during antenatal care in South Asia
Source: Matern Child Nutr. 2021 Feb 7;17(3):e13153. doi: 10.1111/mcn.13153 (PMC8189234; doi:10.1111/mcn.13153)
Supplement: Supplementary file 1 — Figure S1: Conceptual framework of factors related to the quality of counselling on maternal nutrition and infant feeding delivered to pregnant women during antenatal care. Figure S2. Example of search terms applied to the titles and abstracts of articles Figure S3. Flow chart of the study selection process for eligible studies. Table S1: Indicators† of counselling quality studied by each of the included studies that examined the quality of counselling on maternal nutrition and infant feeding during antenatal care† Table S2: Indicators† of counselling quality studied by each of the included studies that examined the effectiveness of programs, interventions, and approaches to improve quality of counselling on maternal nutrition and infant feeding during antenatal care [file MCN-17-e13153-s001.docx]

**SUPPLEMENTARY APPENDIX**

Figure S1: Conceptual framework of factors related to the quality of counseling on maternal nutrition and infant feeding delivered to pregnant women during antenatal care.


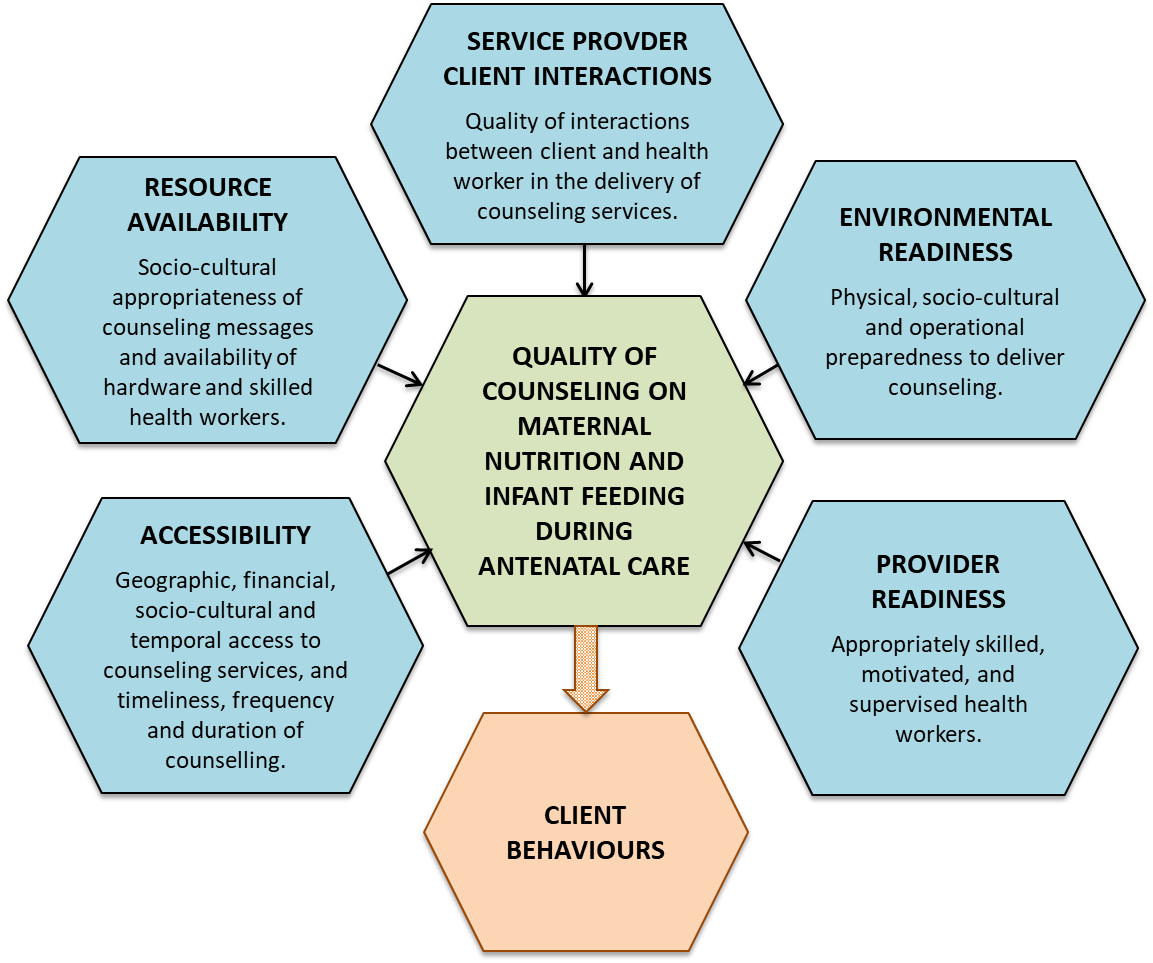


Figure S2. Example of search terms applied to the titles and abstracts of articles

Afghan* or Bangladesh* or India* or Nepal* or Pakistan* or “South Asia* or “southern asia*

pregnan* OR gestat* OR perinatal OR parturient OR mother* OR matern* OR woman OR women OR female

prenatal Care OR perinatal care OR antenatal care OR antenatal counseling OR antenatal visit* OR maternal health services OR support OR counsel* OR program OR educat* OR advise OR advice

maternal nutrition OR infant and young child feeding OR child nutrition OR breastfe* OR breast fe* OR complementary fe* OR diet quality

Figure S3. Flow chart of the study selection process for eligible studies.

Full-text papers screened for eligibility

(n=168)

Full-text articles

not eligible

(n=147)

Full-text not accessible†

(n=9)

Unique records identified through database search and hand search

(n=924)

Excluded after title and abstract review

(n=756)

Included studies

(n=12, 3 studies on quality and 9 studies on effectiveness)

†The authors made every effort available to obtain these articles through online platforms, the Cornell University library and Cornell University’s Interlibrary Loan services; however, the full-text was not accessible for nine papers published in the Indian Journal of Maternal and Child Health (1992-93), Mymensingh Medical Journal (2011), Journal of the College of Physicians and Surgeons Pakistan (2006), and Current Diabetes Review (2016).

Table S1: Indicators† of counselling quality studied by each of the included studies that examined the quality of counseling on maternal nutrition and infant feeding during antenatal care†

| **Study** | **A2** | **A5** | **B1** | **B2** | **B3** | **D2** | **D3** | **D4** | **E1** | **E3** | **E4** | **E6** | **E7** |
| --- | --- | --- | --- | --- | --- | --- | --- | --- | --- | --- | --- | --- | --- |
| Avula et al. (2015) |  |  |  |  | X |  |  |  |  | X |  |  |  |
| Dhandapany et al. (2008) |  |  |  |  |  |  |  |  |  | X |  |  |  |
| Dykes et al. (2012) |  |  |  | X |  | X |  |  |  |  |  | X |  |
| Huda et al. (2018) | X | X |  | X |  |  |  |  |  |  |  |  | X |
| Mahar et al. (2012) | X | X |  |  |  |  |  |  |  | X |  |  |  |
| Majrooh et al. (2014) |  |  |  |  |  |  |  |  |  | X |  |  |  |
| McPherson et al. (2010) |  |  | X | X |  |  | X | X |  |  |  | X |  |
| Pricilla et al. 2018 |  |  |  |  |  |  |  |  | X | X | X |  |  |
| Singh et al. (2012) |  | X |  |  |  |  |  |  |  | X |  |  |  |

†See Table 1 for indicator list

Table S2: Indicators† of counselling quality studied by each of the included studies that examined the effectiveness of programs, interventions, and approaches to improve quality of counseling on maternal nutrition and infant feeding during antenatal care

| **Study** | **A2** | **B3** | **D1** | **D2** | **D4** | **E1** | **E3** |
| --- | --- | --- | --- | --- | --- | --- | --- |
| Baqui et al. (2006) |  | X |  |  |  |  | X |
| Nguyen et al. (2017) |  |  |  |  |  | X |  |
| Nguyen et al. (2017) | X | X | X | X | X |  | X |

†See Table 1 for indicator list
